# Encapsulation of Oleuropein in Nanostructured Lipid Carriers: Biocompatibility and Antioxidant Efficacy in Lung Epithelial Cells

**DOI:** 10.3390/pharmaceutics12050429

**Published:** 2020-05-06

**Authors:** Amaia Huguet-Casquero, Maria Moreno-Sastre, Tania Belén López-Méndez, Eusebio Gainza, Jose Luis Pedraz

**Affiliations:** 1 NanoBioCel Group, Laboratory of Pharmaceutics, University of the Basque Country (UPV/EHU), School of Pharmacy, Paseo de la Universidad 7, 01006 Vitoria-Gasteiz, Spain; amaiahuguetc@gmail.com (A.H.-C.); maria.moreno@ehu.eus (M.M.-S.); tblopez01@gmail.com (T.B.L.-M.); 2 Biosasun S.A., Iturralde 10, Etxabarri-Ibiña, 01006 Zigoitia, Spain; eusebioggg@hotmail.com; 3 Biomedical Research Networking Center in Bioengineering, Biomaterials and Nanomedicine (CIBER-BBN), 01006 Vitoria-Gasteiz, Spain

**Keywords:** oleuropein, polyphenols, nanoparticle, natural antioxidant, nanotechnology, olive oil

## Abstract

Oxidative damage has been linked to a number of diseases. Oleuropein (OLE), a natural occurring polyphenol from olive leaves (*Olea europaea L.*), is known to be a potent antioxidant compound with inherent instability and compromised bioavailability. Therefore, in this work, nanostructured lipid carriers (NLCs) were proposed for OLE encapsulation to protect and improve its antioxidant efficacy. The lipid matrix, composed of olive oil and Precirol, was optimized prior to OLE encapsulation. The characterization of the optimized oleuropein-loaded NLCs (NLC-OLE) showed a mean size of 150 nm, a zeta potential of −21 mV, an encapsulation efficiency of 99.12%, sustained release profile, and improved radical scavenging activity. The cellular in vitro assays demonstrated the biocompatibility of the NLCs, which were found to improve and maintain OLE antioxidant efficacy in the A549 and CuFi-1 lung epithelial cell lines, respectively. Overall, these findings suggest a promising potential of NLC-OLE to further design a pulmonary formulation for OLE delivery in lung epithelia.

## 1. Introduction

Reactive oxygen species (ROS) have an essential role in normal cell function and signaling. However, their overproduction leads to an oxidative stress status that ultimately contributes to the development of several pathological events such as inflammation, fibrosis, genotoxicity, and carcinogenesis. As a result, oxidative stress has been linked to more than 50 diseases involving several human organs [1,2,3,4]. Among them, the respiratory tract is one of the main sites for the presence of prooxidant molecules. Moreover, clinical evidence suggests that many lung diseases are associated with a reduced antioxidant defense, together with an increased accumulation of ROS [5]. Although current strategies toward oxidative damage have been focused on the use of small ROS scavenger drugs (i.e., Edaravone, N-acetylcysteine, Cerovive^®^) their toxicity, together with their low clinical efficacy, has been discouraging [5,6]. Given this, natural antioxidants have emerged as alternative or even complementary candidates with minimized toxicity.

Extracted from olive leaves, oleuropein (OLE) is widely known for its potent antioxidant efficacy which, interestingly, seems to be the basis for its pleiotropic pharmacological activities: hypoglycemic [7], antiviral [8], antimicrobial [9], platelet anti-aggregant [10], anticancer [11,12], hypolipidimic [13], and anti-inflammatory [14,15], among others. Even the European Medicines Agency (EMA) as well as the European Food Safety Agency (EFSA) have made their own assessment reports about the health-promoting properties of this natural compound in human health [16,17]. Despite its promising properties, the therapeutic use of OLE is limited by its poor stability against environmental (light, oxygen, temperature) and human biological conditions (pH, enzymes) [18], resulting in compromised bioactivity, bioaccessibility, and bioavailability [19]. Along with these limitations, the lack of target specificity hampers the therapeutic application of OLE.

In this context, micro and nanoencapsulation technologies have been proposed as promising strategies [20]. Currently, some encapsulation methods for *Olea europaea* leaf extracts have been described such as W/O nanoemulsions and W/O/W double emulsions [21,22,23], spray-drying [24,25], formation of inclusion complexes with cyclodextrin [26], electrostatic extrusion [27], biopolymer complexes/double emulsions [21], and liposomes [28]. However, they have some limitations such as the use of organic solvents, tedious and time consuming manufacturing processes, high particle sizes, and low yield (for spray drying). Furthermore, a low amount of OLE is normally encapsulated in the final formulation (~1–2% *w/w*) and there is a lack of tests ensuring the preservation of OLE antioxidant activity in biological systems. Given this scenario, lipid nanoencapsulation and particularly, lipid nanostructured carriers (NLCs) offer special advantages: scale-up feasibility, long-term stability, high drug loading, sustained release, good biocompatibility, biodegradable properties, and most importantly, the increased local deposition of the drug [29,30,31,32]. Moreover, the lipid excipients used for NLC formulation have documented biological anti-inflammatory effects in several disease models, which might result in a synergistic effect with the encapsulated compound [33,34]. NLCs are made up of a lipid core formed by a mixture of solid and liquid lipids stabilized by surfactants and offer the possibility of incorporating both lipophilic and hydrophilic drugs [35]. The selection of the encapsulating material is critical in the design of the nanoparticles [36]. In this work, Precirol and olive oil were chosen to form the lipid core due to its efficacy in sustained release formulations and its ability to reduce the possible cytotoxicity effect of residual surfactants that might be present in the final nanoformulation, respectively [37].

Hence, the goal of this work was to elaborate OLE-loaded NLCs to modulate the antioxidant activity of the encapsulated compound in lung epithelial cells. With the aim to obtain a nanoformulation with suitable physico-chemical properties, a preformulation study was conducted varying the amount of liquid and solid lipids of the nanoparticle matrix. The most promising nanoformulation was chosen for the evaluation of OLE encapsulation, in vitro release profile, thermal behavior, and chemical antioxidant activity. Finally, the biocompatibility of OLE-loaded NLCs as well as their cellular antioxidant efficacy were investigated in three lung epithelial cell models.

## 2. Materials and Methods

### 2.1. Materials

#### 2.1.1. Chemicals

Precirol^®^ ATO 5 (glycerol distearate) was a kind gift from Gattefosé (Saint-Priest, France). Polysorbate and Tween^®^ 80 were purchased from Panreac Química (Castellar del Vallès, Barcelona, Spain). Organic extra virgin olive oil was donated by Biosasun S.A. (Álava, Spain). Poloxamer 188 and 2,2-diphenyl-1-picrylhydrazyl (DPPH) were kindly provided by Merck (Darmstadt, Germany). D-trehalose anhydrous was purchased from ACROS Organics™ (Geel, Belgium). OLE (>80%) was donated by Nonaherbs Bio(Tech) (Xi’an, China). Ascorbic acid was purchased from Sigma-Aldrich Chemicals (St. Louis, MO, USA). The ultrapure water was from a Milli-Q Water System. Other chemicals were all analytical grade.

#### 2.1.2. Cell Culture Reagents

A549 (ATCC^®^ CCL-185™), CuFi-1(ATCC^®^ CRL-4013™) and NuLi-1 (ATCC^®^ CRL-4011™) cells were bought from the American Type Culture Collection (ATCC; Manassas, VA, USA). Roswell Park Memorial Institute (RPMI) 1640 medium without phenol red, N-2-hydroxyethylpiperazine-N-2-ethane sulfonic acid (HEPES), penicillin-streptomycin (PEST), inactivated fetal bovine serum (FBS), Dulbecco’s Phosphate Buffered Saline (DPBS) and trypsin-EDTA (0.5%) without phenol red were purchased from Gibco™ (Life Technologies, Madrid, Spain). Serum-free Bronchial Epithelial Growth Medium (BEGM Bullet Kit; CC-3170) made of BEBM basal medium and SingleQuot additives; Airway Epithelial Cell Basal Medium and Bronchial Epithelial Cell Growth Kit additives were purchased from Lonza (Clonetics, Lonza, Walkersville Inc., Walkersville, MD, USA). Dimethyl sulfoxide (DMSO) was purchased form Scharlau (Madrid, Spain). Human Placental Collagen Type IV (Sigma Cat. No. C-7521) and Cell Counting Kit-8 (CCK-8) were bought from Sigma-Aldrich (Saint Louise, MO, USA). OxiSelect™ Cellular Antioxidant Cell Kit was bought from Quimigen (Madrid, Spain) to Cell Biolabs, Inc. (San Diego, CA, USA).

### 2.2. Preparation of Nanostructured Lipid Carriers (NLC)

#### 2.2.1. Blank-Nanostructured Lipid Carriers (NLCs)

Nanostructured lipid carriers, NLCs, were elaborated by the hot melt homogenization method as previously described by our research group [38] with few modifications. Briefly, Precirol ATO^®^ 5 (solid lipid; melting point: 56 °C) and olive oil (liquid at room temperature (RT)) were chosen to form the lipid core. Several solid–liquid lipid proportions ranging from 10:90 to 90:10 were employed for each batch manufacturing to study their effect in formulation parameters. This lipid phase was melted 5 °C above the solid lipid (Precirol ATO^®^ 5) melting point until a clear and homogeneous phase was obtained. The aqueous phase was prepared by dispersing 1.3% (*w/v*) of Tween^®^ 80 and 0.66% (*w/v*) of Poloxamer 188 in Milli-Q water and heating to the same temperature as the lipid phase. Straightaway, the hot aqueous phase was added to the melted oily phase, and then sonicated for 30 s at 50 W (Branson Sonifier 250, Danbury, CT, USA). The formed nanoemulsion was maintained under magnetic stirring during 10 min at RT and stored for 2 h at 4 °C to allow the re-crystallization of the lipids and NLC formation. Then, the particles were collected using a 100-kDa molecular weight cut-off centrifugal filter unit (Amicon, “Ultracel-100k”, Millipore, Spain) at 2500 rpm for 10 min and washed three times with MillliQ water. All of the nanoparticles prepared were freeze-dried for 36 h (Telstar Lyobeta freeze-dryer, Terrasa, Spain). Prior to the lyophilization process of the resulting NLC suspension, a solution of a cryoprotectant (trehalose (15% *w/w*)) was added to the collected nanoparticles.

#### 2.2.2. Oleuropein (OLE)-Loaded NLCs (NLC-OLE)

From the developed formulations, the lipid matrix accomplished with the best physico-chemical characteristics was selected for OLE encapsulation studies. OLE-loaded NLCs were prepared as described above, but adding the corresponding volume of a saturated solution of OLE in purified water (0.66 mg/mL). As OLE is hydrophilic and slightly thermosensitive, it was blended in the molten lipid phase just prior to the addition of the aqueous phase and the following sonication process. The targeted loading of OLE in NLCs were 30% (NLC-OLE30), 40% (NLC-OLE40), and 50% (NLC-OLE50) (*w/w*).

### 2.3. Characterization of Lipid Nanoparticles

#### 2.3.1. Size and Zeta Potential

The mean particle size (Z-average diameter) was measured by dynamic light scattering (DLS) and zeta potential was determined through Laser Doppler micro-electrophoresis (Malvern^®^ Zetasizer Nano ZS, Model Zen 3600; Malvern instruments Ltd., Malvern, UK). Prior to the measurements, nanoparticles were dispersed in Milli-Q water (pH 5.6) at optimal intensity. For zeta potential, the measured electrophoretic mobility was converted into zeta potential through Smoluchowski approximation. Each assay was performed in triplicate before and after the nanoparticles’ lyophilization and data are presented as mean ± S.D.

#### 2.3.2. Thermogravimetric Analysis

To assess the moisture content of freeze-dried nanoparticles, a thermogravimetric analysis was performed (NETZSCH, STA 449 F1 Jupiter^®^, Barcelona, Spain). Samples (NLC-empty, NLC-OLE30, NLC-OLE40, and NLC-OLE50) were heated from 25 to 200 °C at a heating rate of 5 °C/min under a nitrogen atmosphere. Results are expressed as water content (%).

#### 2.3.3. High-Performance Liquid Chromatography (HPLC) Method

Chromatographic analysis was performed using Alliance 2795 Waters equipment coupled to an UV/Vis detector. Chromatographic separation was performed in a Zorbax Eclipse Plus^®^ C18 column (250 mm × 4.6 mm I.D.; 5.0 µm; Agilent Technologies, Wilmington, DE, USA) column. The mobile phase consisted of acetonitrile:water (25:75 (*v/v*)) and pH was adjusted with ortophosphoric acid to 3. The system was operated isocratically at a flow rate of 1.0 mL/min, and detection was performed at 230 nm. The retention time of OLE was 7.6 min at room temperature and the total run time of HPLC analysis was 10 min. Prior to injection, samples (10 μL) were diluted in water and filtered (0.45 µm). The method was validated in terms of linearity, accuracy, and specificity over the range of expected concentrations. A linear correlation was observed in the concentration range of 6.20–420 μg/mL, with a coefficient of determination *r*^2^ = 0.999 and RSD < 2%. The limit of detection and limit of quantification were 0.2 µg/mL and 1.0 µg/mL, respectively. The method was specific for OLE and no interfering peaks were observed near its retention time.

#### 2.3.4. Encapsulation Efficiency (EE)

Encapsulation efficiency of OLE into NLCs was determined indirectly by measuring the free OLE (non-encapsulated OLE) in the supernatant obtained after the filtration/centrifugation process. OLE concentration was quantified by the explained HPLC method. Considering the initial amount of OLE added to each formulation, the EE was calculated as:

(1) $$EE\;\left(\%\right)=\left(\frac{Total\;amount\;of\;OLE-Amount\;of\;free\;OLE}{Total\;amount\;of\;OLE}\right)\times 100,$$

#### 2.3.5. Microscopy Analysis

NLC surface characteristics and morphology were examined under transmission electron microscopy (TEM, JEOL JEM-1400 Plus a 120 kV, Peabody, MA, USA). For this purpose, lyophilized samples were suspended in Milli-Q water at an optimal concentration of 4 mg/mL and sonicated in a water bath for one minute. Then, samples were placed on a carbon grid and treated with negative staining uranyl acetate (2%) for particle visualization. Images were captured with a digital camera sCMOS (Hamamatsu, Hawthorne, CA, USA).

#### 2.3.6. In Vitro Drug Release Studies

In order to obtain qualitative and quantitative information on OLE release from NLCs, in vitro drug release studies were conducted using Quix-Sep Micro Dialyzers (Membrane Filtration Products Inc., Seguin, TX, USA) at 37 °C under magnetic stirring in phosphate buffer saline (PBS, pH 7.4). A dialysis regenerated cellulose tubular membrane with a molecular weight cut-off (MWCO) between 12,000 and 14,000 Da was used. First, cellulose membranes were soaked in the dissolution medium (PBS) for 12 h prior to its use to ensure thorough wetting of the membrane before placing it in a Quix-Sep cell. To carry out this study, the NLC-OLE30, NLC-OLE40, and NLC-OLE50 suspensions (25 mg/mL) were placed in the cell system, which was immersed in 30 mL of PBS preheated solution (pH 7.4) as the dissolution medium. At fixed time intervals up to 27 h, dissolution mediums were removed from the incubation and replaced with new preheated PBS medium. Samples were kept at 4 °C until they were analyzed by HPLC (see Section 2.3.3). The release study was carried out under proper sink conditions. Results were expressed as percentage of OLE released compared to the total compound encapsulated in the nanoformulation. Experiments were run in triplicate for each point of release kinetics. To further study OLE release kinetics and mechanism, the obtained cumulative release data were computed with the DDsolver program and fitted to several kinetic models: zero order, first order, Higuchi, Baker–Londslade, Hixson–Crowell, Hopfenger, and Korsmeyer–Peppas [39]. Regression coefficient (r^2^) was calculated to determine the best-fit model.

#### 2.3.7. Differential Scanning Calorimetry (DSC)

The thermal behavior of the freeze dried NLCs was studied using differential scanning calorimetry (DSC-50, Shimadzu, Japan). One to two milligrams of each sample was weighed and placed on an aluminum pan and crimped. The samples were heated from 25 °C to 350 °C at the rate of 10 °C/min. Pure solid lipid powder (Precirol^®^ ATO5), OLE powder, pure solid surfactant (Poloxamer^®^ 188), and cryoprotectant (trehalose anhydrous) were also subjected to DSC analysis to gather additional information. The crystallinity index (CI) of NLCs was calculated from the enthalpy of fusion using the following equation:

(2) $$CI\;\left(\%\right)=\frac{\mathrm{Enthalpy}\;\mathrm{NLC}\;\left[J/g\right]}{\mathrm{Enthalpy}\;\mathrm{solid}\;\mathrm{lipid}\left[J/g\right]}\times 100,$$

#### 2.3.8. Radical Scavenging Activity Assessment by the 2,2-Diphenyl-1-Picrylhydrazyl (DPPH) Method

Radical scavenging activity of the nanoparticles was investigated spectrophotometrically, analyzing their ability of scavenging DPPH radical [40]. Briefly, a solution of DPPH was prepared with a concentration of 0.1 mM in absolute ethanol. Freeze-dried NLC-OLE50 was diluted in PBS to obtain a final concentration of 1.25 mg/mL and they were left to release the drug for 24 h in the same conditions as described in Section 2.3.6. Equivalent concentrations of OLE (0.2 mg/mL) and NLC-empty (1.25 mg/mL) were also prepared in PBS and assayed in the same conditions. Ascorbic acid (0.2 mg/mL) was used as the method control. A total of 500 µL of each sample was added to 3300 µL of DPPH-ethanol solution. The reaction mixture was incubated for 60 min protected from light on a shaker at 37 °C. After that, the absorbance of the reaction solutions was recorded at 517 nm by UV–Vis spectrophotometry (6705 UV/Vis Spectrophotometer JENWAY, Fisher Scientific SL, Madrid, Spain). DPPH radical scavenging activity was calculated according to the following equation:

(3) $$DPPH\;scavenging\;activity\;\left(\%\right)=\left(\frac{Abs\;control-Abs\;sample}{Abs\;control}\right)\times 100,$$

where *Abs_sample_* is the absorbance of the DPPH solution after reacting with the sample, and *Abs_control_* is the absorbance of blank 0.1 mM DPPH solution. Ethanol was used as the blank. All measurements were carried out in triplicate and the results are expressed as mean ± standard deviation.

### 2.4. Cell Experiments

#### 2.4.1. Cell Culture

Human lung adenocarcinoma epithelial cells (A549) were grown and maintained in RPMI 1640 medium (pH 7.4) supplemented with 10% (*v/v*) inactivated FBS, 1% PEST, and 1% HEPES, without phenol red and incubated at 90% humidity, 5% (*v/v*) CO_2_ atmosphere at 37 °C. Cells were allowed to grow until 90% of confluence. Then, they were trypsinized (Trypsin-EDTA) and seeded in plates for each experiment. Cystic fibrosis (CuFi-1) cell line, derived from a CF human bronchial epithelium homozygous for the CFTR ΔF508 mutation, was grown and maintained in serum-free BEGM medium at 37 °C, 90% humidity and 5% CO_2_. All culture-flasks were pre-coated with 60 µg/mL solution of Human Placental Collagen Type IV at least 18 h in advance, then air-dried and rinsed 2–3 times with DPBS. Cells were allowed to grow until 80% of confluence, trypsinized (Trypsin-EDTA) and seeded in plates for each experiment. Plates were also pre-treated with collagen as explained before. Human normal bronchial epithelial (NuLi-1) cells were grown and maintained under the same conditions as CuFi-1 cell line but in serum-free Airway Epithelial Cell Basal medium supplemented with Bronchial Epithelial Cell Growth Kit additives.

#### 2.4.2. Cell Viability Studies

Biocompatibility of empty nanoparticles (NLC-empty) and OLE-loaded nanoparticles (NLC-OLE50) was evaluated in the A549, CuFi-1, and NuLi-1 cell lines. Each cell line was plated in 96-well microtiter plates at a density of 10,000 cells/well (A549) and 15,000 cells/well (NuLi-1, CuFi-1) in a final volume of 100 µL of the corresponding cell medium. Cells were treated with NLC-OLE50 suspended in cell medium at concentrations ranging from 14.45 to 462.5 µM (in terms of encapsulated OLE) and the equivalent amounts of NLC-empty, for 24 h at 37 ± 2 °C, 90% humidity, and 5% CO_2_. Controls were set with dimethyl sulfoxide (DMSO) as the negative or death control, and medium without formulation as the positive control. Free OLE was assayed for comparison. After 24 h of incubation, cell viability was determined with the CCK-8. Cells were washed with sterile DPBS and then 10% of CCK-8 in medium was added to each well and incubated in a wet chamber for 4 h at 37 ± 2 °C and 5% CO_2_. The resulting colored solution was quantified using a microplate reader (Infinite1 200 PRO, Tecan, Männedorf, Switzerland). The spectrophotometric absorbance was measured at 450/650 nm wavelength. Results were calculated in relation to the untreated cells (~100% viability) and are expressed as the percent of cell viability ± standard deviation of the values collected from three separate experiments performed in triplicate for each sample and each cell line.

#### 2.4.3. Cellular Antioxidant Activity (CAA) Assay

Cellular ROS scavenging activity of NLC-OLE50 was measured using the OxiSelect™ Cellular Antioxidant Activity Assay Kit in the A549, CuFi-1 and NuLi-1 cell lines. 2′,7′-dichlorodihydrofluorescin diacetate (DCFH-DA) was used as the fluorogenic probe. Cells were seeded as previously explained in clear bottom black polystyrene 96-well plates for 24 h. After that, all media was removed and washed gently with DPBS w/o calcium and magnesium three times. Cells were then treated with OLE, both free and nanoencapsulated, at the following concentrations: 115 µM, 231.2 µM, and 462.5 µM. After 24 h of sample incubation at 37 °C, 90% humidity, and 5% CO_2_, the CAA assay was carried out. Briefly, wells were washed with sterile DPBS and then 50 μL of DCFH-DA solution was added. Plates were incubated for 60 min at 37 °C, 90% of humidity, and 5% CO_2_ in order to allow the cell-permeable fluorogenic probe dye (DCFH-DA) to diffuse into the cells. After incubation, all solutions were removed and wells were washed three times with DPBS. After addition of 100 μL of 2,2′-azobis (2-amidinopropane) dihydrochloride (ABAP) solution, as the free radical initiator, fluorescence was read at 37 °C for 60 min with 5 min intervals with the excitation wavelength 480 nm and emission wavelength of 530 nm.

Each plate included cell control wells (cells without any treatment), negative control wells (cells pretreated only with DPBS and DCFH-DA), and positive control wells (cells pretreated with ABAP and DCFH-DA). Quercetin was used as a standard in each experiment and was added following provider instructions, just prior to the addition of DCFH-DA with the aim to validate the assay in the selected cell models. Absence of green fluorescence in the studied samples was confirmed before the assay. Each assay was carried out in triplicate for each cell line.

### 2.5. Statistical Analysis

All results were expressed as the mean ± standard deviation (SD) unless otherwise stated. All statistical analyses were calculated using GraphPad Prism 6 Statistics software (San Diego, CA, USA) and *p* < 0.05 was considered as significant. A multiple-sample and two-sample *t* test with unequal standard deviations was used to verify the significant difference between data in cellular antioxidant and DPPH assays, respectively. For cell viability results, a two-way ANOVA was run.

## 3. Results and Discussion

### 3.1. Optimization and Physico-Chemical Characterization of Nanoparticles: Particle Size, Morphology, and Zeta Potential

In the first step of this work, NLCs were prepared through the hot melt emulsification method followed by ultrasonication (HME-Us), as previously described by our group [38]. The rationale for using this technique relies on its versatility and easy scale-up. HME-Us have gained a lot of importance after the Food and Drug Administration encouraged the use of continuous processes among the pharmaceutical industry [41] and overcome most of the limitations offered by the conventional micro- and nanoemulsification techniques. Avoidance of organic solvents, shorter and lower steps of the process as well as the increased homogeneous spreading of the particles are some of the advantages of this method [42,43]. Altogether, this makes HME-Us an industrially and environmentally friendly technique that has largely been applied in the preparation of NLCs for multiple applications [44,45].

Unlike other lipid nanoparticles, the lipid core of NLCs is composed by the blend of a liquid lipid and a solid lipid. The presence of this liquid lipid leads to a more amorphous matrix and a less crystalline state of the carrier, which results in the accommodation of a higher number of drug molecules compared to other lipid nanoformulations [45]. In this work, Precirol ATO 5 as the solid lipid and olive oil as a natural liquid lipid were chosen for the lipid core formulation. Precirol ATO 5 has largely been used as a component of lipid matrix for sustained release formulations and has proven to be effective in the formulation of NLCs for pulmonary delivery [30,38,46]. For liquid lipids, the medium chain triglycerides, known under the brand name Miglyol 812, are the most commonly employed. However, we selected olive oil as it is thought to reduce the possible cytotoxicity effect of residual surfactants that might be present in the final nanoformulation [37]. Accordingly, we employed an aqueous phase composed of the minimum amount of Tween 80 (1.3%, *w/v*) and Poloxamer 188 (0.66%, *w/v*), which led to a nanoformulation with good physico-chemical stability as previously demonstrated by our group [47,48]. Particle size and superficial charge are important parameters in NLC development. Whilst particles sizes below 500 nm are generally thought to escape from phagocytosis by macrophages [49], a negative superficial charge of around −20 mV is generally correlated with good physical stability of the nanoparticle dispersion [50] as well as their attraction to the positively charged proteins from damaged tissues [45,51]. Finally, aimed to improve NLC stability, 15% (*w/w*) of trehalose was chosen as a cryoprotectant as it was found to be the most suitable one for the lyophilization process [38].

Therefore, the aim of this work was to obtain, through the HME-Us method, a NLC formulation with the highest amount of olive oil as the liquid lipid and adequate physico-chemical characteristics. To attain this purpose, various solid–liquid lipid ratios from 10:90 (formulation 1) to 90:10 (formulation 9) were tested. Aqueous phase composition was always the same for all formulations. As summarized in Table 1, it seemed that the higher the olive oil content, the smaller the particle size, which was then confirmed by TEM micrographs (see Supplementary Materials). These results were in line with other authors, who also reported a decrease in the particle size of NLCs when the liquid lipid amount was increased [52,53,54]. Nevertheless, all formulations displayed adequate sizes between 100 and 200 nm. Due to the dissociation of protons from the carboxylic groups of Precirol after dilution in deionized water (pH 5.6), NLCs exerted a negative superficial charge, which was found to be around −20 mV for all nanoformulations and thus all of them might have good physical stability [55,56]. In contrast, olive oil content significantly affected the freeze-drying process of the NLCs. Particularly, we found that the amount of olive oil in the lipid core should be ≤50% (*w/w*) to successfully achieve a lyophilized product (Supplementary Materials, Section S1).

Considering all the aforementioned, formulation 6, with an adequate particle size (~140 nm) and superficial charge (~−22 mV) as well as the highest amount of olive oil in the lipid core (40%, *w/w*) that allowed for a successful lyophilization process, was selected for the forthcoming loading studies.

### 3.2. Physico-Chemical Characterization of OLE-Loaded Nanoparticles

#### 3.2.1. Particle Size, Z-Potential, Encapsulation Efficiency, Morphology, and Moisture

**Figure 1:**
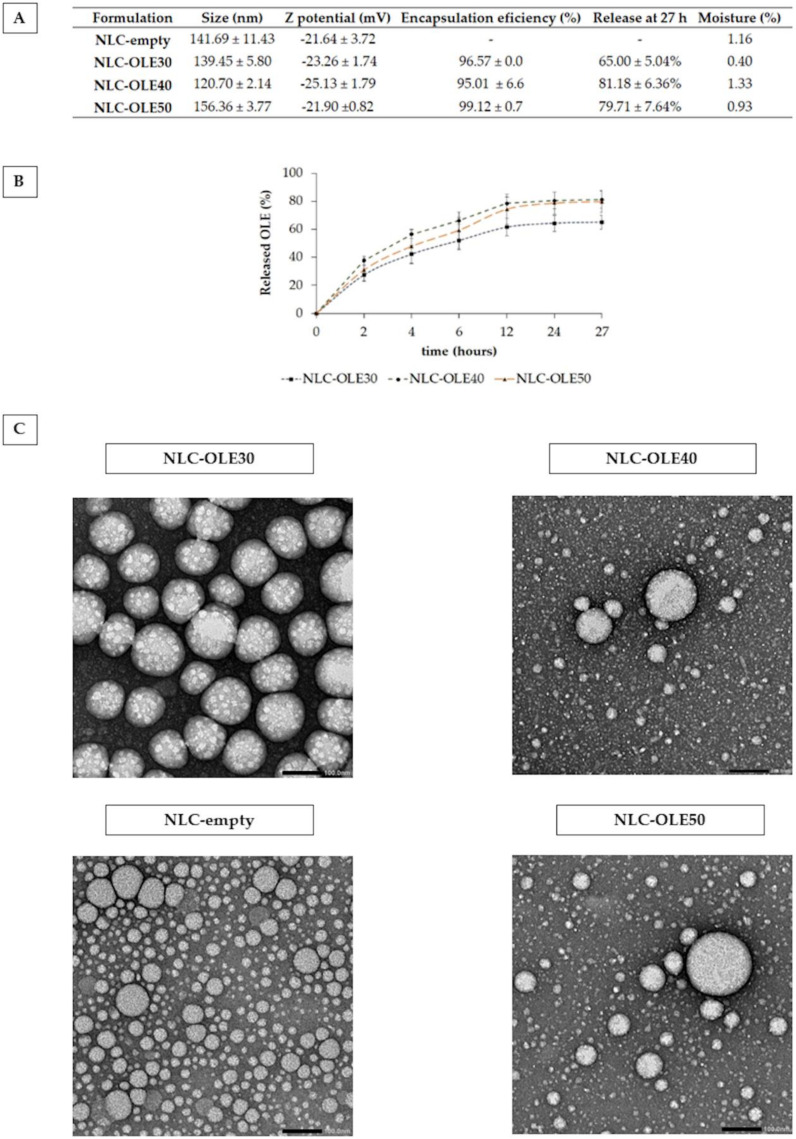
Physico-chemical characterization of freeze-dried nanostructured lipid carriers: empty (NLC-empty) and oleuropein (OLE) loaded, with a targeted OLE loading of 30% (NLC-OLE30), 40% (NLC-OLE40) and 50% (NLC-OLE50) (*w/w*). (**A**) Particle size, Z potential, encapsulation efficiency, total released OLE and moisture content of NLCs. (**B**) In vitro release of OLE from developed NLCs. (**C**) TEM micrographs of OLE-loaded and empty nanoparticles. The scale bar indicates 100 nm.

As shown in Figure 1A, OLE-loaded NLCs exhibited sizes of around 140 nm (NLC-OLE30), 120 nm (NLC-OLE40), and 150 nm (NLC-OLE50). For the zeta potential, no significant differences were found after OLE incorporation into the lipid matrix, indicating that physical stability of the nanoparticles was not disturbed by OLE loading. With regard to the water content, similar low moisture values ranging from 0.4% (NLC-OLE30) to 1.33% (NLC-OLE40) were found. As depicted by the TEM micrographs (Figure 1B), OLE-loaded nanoparticles displayed a uniform and rounded shape. According to the 100 nm scale-bars in the images, the mean diameter was found to be less than 200 nm in all cases, validating the results obtained with DLS techniques (Figure 1A). Finally, the entrapment efficiency of OLE (Figure 1A) was above 95% in all cases, and thus, the developed NLCs were validated as an adequate delivery system for future applications.

#### 3.2.2. In Vitro Drug Release

Results from the performed in vitro release studies, at physiological pH and temperature (pH 7.4 and 37 °C, respectively), are reported in Figure 1C as the percentage of OLE released over time. A sustained release of the olive polyphenol was detected in all cases up to 27 h, which might suggest that OLE was protected inside the core of the lipid carries. For the NLC-OLE40 and NLC-OLE50 formulations, a total OLE-release of ~80% was found by the end of the study (27 h). However, NLC-OLE30 showed a significantly lower percentage of released OLE (65% after 27 h). It is known that the release profile of OLE could have a significant effect on its antioxidant and bioactive properties. Therefore, aimed to ensure that the obtained release patterns of NLCs were sustained as well as to describe the release mechanism of OLE from the developed NLC matrices, mathematical kinetic models were applied to the experimental data obtained in the release assay (Table 2) [57,58]. The regression coefficient value (r^2^) was used to choose the model that best fitted the data. In this work, Korsmeyer–Peppas showed the highest r^2^ for all nanoparticle formulations with r^2^ = 0.977 (NLC-OLE30), r^2^ = 0.998 (NLC-OLE40), and r^2^ = 0.999 (NLC- OLE50), respectively. This type of release has been reported before for some nanostructured lipid carriers [59,60,61]. In addition, the diffusional exponent ‘*n*’ of the Korsmeyer–Peppas model also described the OLE release mechanism, which in this case was in the range 0.46 to 0.59, indicating that OLE was released by an anomalous transport mechanism [58]. These results imply that probably a combination between erosion and diffusion contributes to the release of the olive polyphenol from the lipid matrix of the nanoparticle.

All in all, we demonstrated the sustained release of OLE via a nanocarrier delivery system that could provide the opportunity to maintain prolonged targeted lung exposures to OLE and thus, longer residence time in lung tissue.

#### 3.2.3. DSC

**Figure 2:**
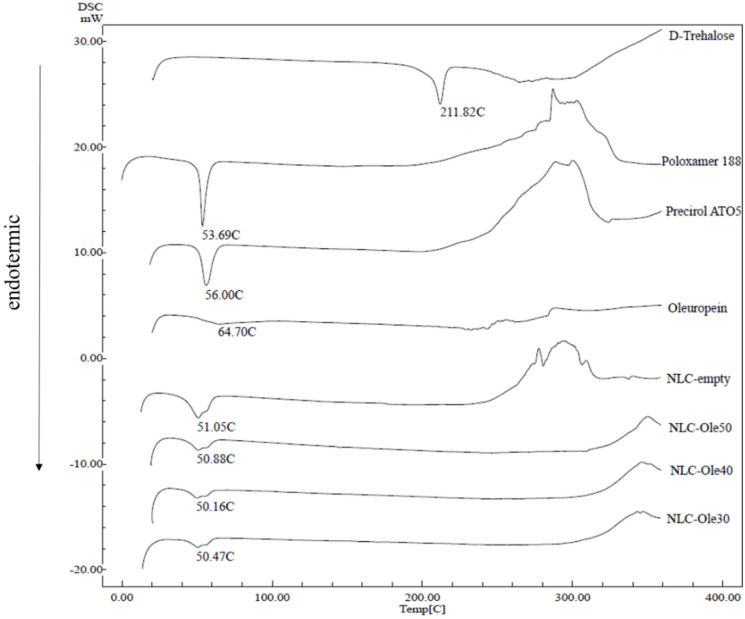
Differential scanning calorimetry (DSC) graphs of pure excipients (D-trehalose, Poloxamer 188, Precirol ATO 5, oleuropein (OLE), developed nanoparticles with OLE (NLC-OLE30, NLC-OLE40, NLC-OLE50), and without OLE (NLC-empty).

Crystallization and thermal behavior of nanoparticles are important properties that determine their utility as drug delivery systems [62,63]. Given this context, DSC thermograms and endothermic events of the bulk solid lipid, bulk OLE, and NLCs (NLC-empty, NLC-OLE30, NLC-OLE40, and NLC-OLE50) were analyzed. Results of the conducted thermal analysis are shown in Figure 2 and Table 3.

Solid lipid Precirol^®^ ATO 5 is known to have a melting range from 50 °C to 60 °C. As shown in Figure 2, bulk Precirol^®^ ATO 5 exhibits a sharp and single endothermic peak at 56.00 °C related to its melting point. Since the diester fraction of the glyceride is apparently the only fraction present in the bulk material, the main modification in which Precirol crystallizes should be the stable β-form, as occurs with most solid lipids. The DSC curve of bulk OLE (Figure 2) exerted a small but broad endothermic event, ascribed to its melting point, at 64.70 °C, which ended up at around 103 °C, followed by its decomposition. This result emphasizes the chemical stability of OLE under the nanoparticles’ manufacturing conditions. Poloxamer 188 showed a sharp melting endothermic peak at 56.69 °C and trehalose anhydrous displayed its melting endotherm at 211.82 °C. For the thermograms of OLE-loaded and unloaded NLCs, they showed two broadening endothermic peaks. The highest temperature endotherms (around 56 °C) can be seen as a shoulder and are similar to the peak of Poloxamer 188. The other endothermic event, around 50 °C, was at lower temperatures than that of the solid lipid. It can be seen that these peaks were broader for NLC-empty. Therefore, there was a clear melting point depression in all NLCs’ thermograms. This phenomenon is generally ascribed to the transformation of the bulk solid lipid into its nanoparticle form as a result of their higher specific surface area or the possible chemical interactions between solid lipid and liquid lipid/or surfactants/or drugs that could take place during the nanoparticle production process and affect crystallization and result in a lower melting enthalpy. Furthermore, the melting peak of OLE (64.7 °C) seemed to be absent in the thermograms of the NLC-OLE formulations, suggesting that the encapsulated olive polyphenol was in an amorphous state. As a result, we assumed that NLCs might offer a greater bioavailability of OLE, as the melting point, an indicator of intermolecular attractive forces, is usually lower for non-crystalline substances.

As expected, the addition of olive oil into the nanoparticle matrix clearly decreased the energy required to melt the lipid (Table 3). On the other hand, Table 3 shows the enthalpy values for bulk Precirol (−150.56 J/g), NLC-empty (−90.21 J/g), NLC-OLE30 −56.22 J/g), NLC-OLE40 (57.15 J/g), and NLC-OLE50 (63.09 J/g). Furthermore, all nanoformulations showed a lower crystallinity index than the bulk solid lipid. Interestingly, OLE-loaded NLCs displayed a greater decrease in both enthalpy and CI values compared to NLC-empty. This energy reduction could be ascribed to the transformation of the solid lipid (Precirol) into a less-ordered metastable β′-form, which leads to a disruption of the crystalline structure and diminishes the CI, which could allow enough space to accommodate OLE molecules.

Thus, the developed NLCs seemed to be a potential carrier for OLE and NLC-OLE50 was selected to continue with the rest of the assays, since this formulation exhibited good physico-chemical characteristics together with the highest OLE amount.

#### 3.2.4. Radical Scavenging Activity by the DPPH Assay

**Figure 3:**
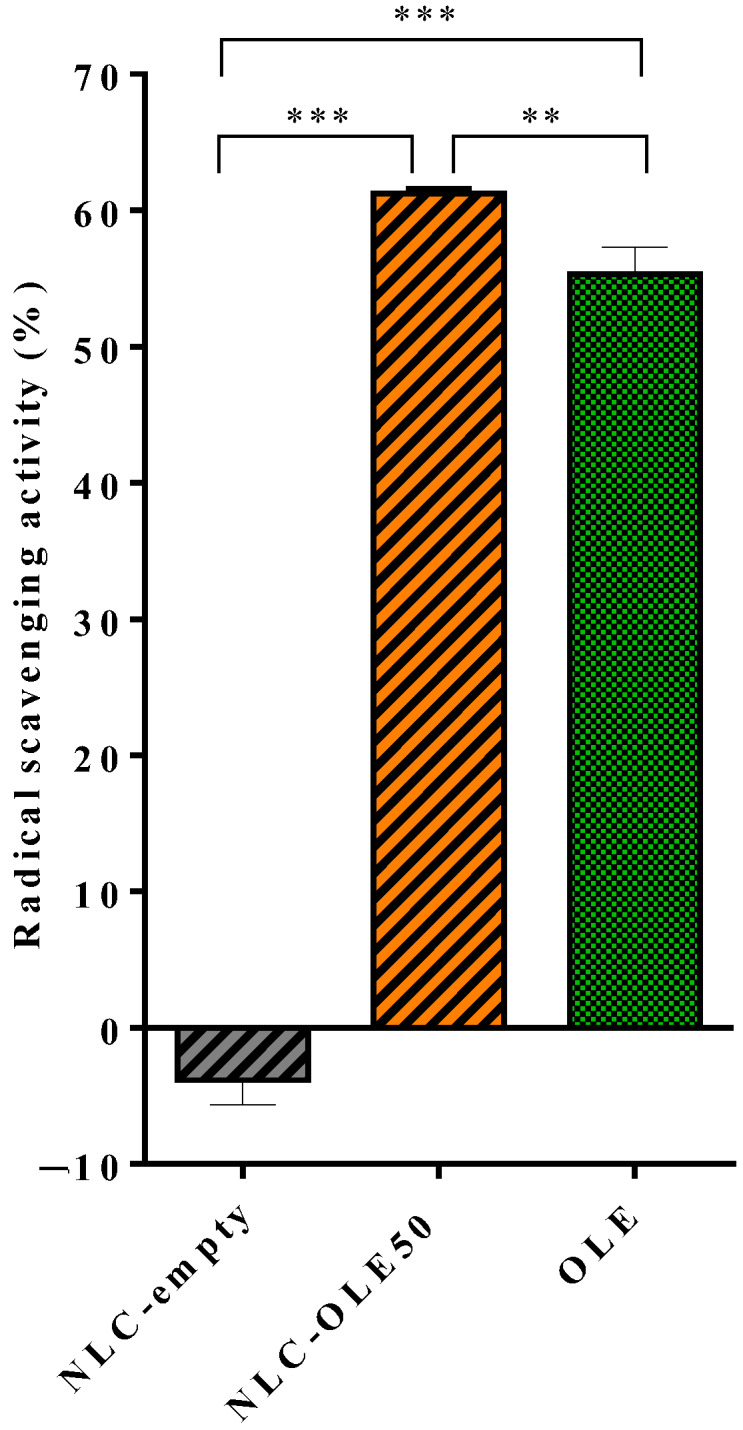
2,2-diphenyl-1-picrylhydrazyl (DPPH) radical scavenging assay of NLC-OLE50 and equivalent amounts of free OLE and NLC-empty. Results are expressed as mean % of DPPH radical scavenging activity compared to the control ± SD; *n* = 3; ** *p* < 0.05, *** *p* < 0.001.

The main bioactivity of OLE is related to its ability to eliminate free radicals and prevent lipid peroxidation, which, in the end, contributes to alleviate the injuries caused by oxidative stress as described elsewhere. Given this, the next step of this work was to verify that the antioxidant activity of OLE was not disturbed by the nanoformulation process, and thus the radical scavenging activity of NLC-OLE50 was assayed. As shown in Figure 3, free OLE had an antioxidant power of 55.33 ± 1.94%, which was significantly enhanced by its incorporation in NLCs (61.22 ± 0.38%). These results could be ascribed to the higher specific area of the nanoparticles for chemical quenching as well as the protection of the polyphenol against external agents into the lipid matrix. Similarly, the encapsulation of other natural compounds such as beta-carotene or quercetin into protein-based and polyvinyl alcohol–based nanoparticles, respectively, and the co-loading of tocopherol and ascorbic acid in nutriosomes have been shown to significantly improve their DPPH radical scavenging activity [64,65,66]. It is worth noting that unloaded-NLCs did not exhibit any radical scavenging activity (−3.84 ± 1.79%) and thus, the lipid excipients of the carrier had no influence on the antioxidant power of NLC-OLE50. Hence, we demonstrated that the nanoencapsulation process through the hot-melt homogenization technique did not decrease OLE activity but improved it.

### 3.3. Cell Experiments

In light of the characterization results, NLC-OLE50 seemed to be a promising carrier for OLE with enhanced antioxidant activity and thus we continued to study its efficacy in lung epithelial cell models. Several authors have reported micro- and nanoencapsulation methods for olive leaf extracts but to the best of our knowledge, there is a lack of studies regarding the impact that the formulation process could have on the bioactivity of this polyphenol in a biological system [22,23,52].

#### 3.3.1. Cell Viability Studies

**Figure 4:**
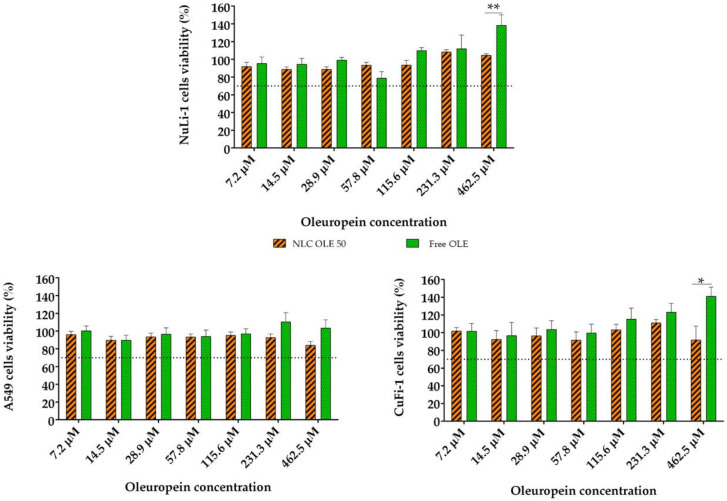
Effect of nanoencapsulated (NLC-OLE50) and free oleuropein (OLE) at different concentrations on the viability of the A549, NuLi-1, and CuFi-1 cell lines among 24 h. The results are given as the mean % of living cells compared to the control ± SD, *n* = 3. * *p* < 0.05, ** *p* < 0.01.

Nanotoxicology has gained special attention during the last decades. The main disadvantage of nanoscale drug delivery systems is their potential cytotoxicity [44]. Generally, nanoparticle toxicity is correlated with the type of excipients and organic solvents employed during the manufacturing process that remain in the final formulation. In this work, we proposed NLCs made up with generally recognized as safe (GRAS) excipients and the use of organic solvents was avoided during their preparation. Moreover, the encapsulated natural compound is known to have low toxicity. Altogether, they lead us to hypothesize that NLC-OLE50 will result in a highly biocompatible formulation with minimum toxicity to the lung epithelium. Therefore, we aimed to prove our hypothesis and thereafter selected the appropriate NLC concentration for cellular oxidative stress studies, the biocompatibility of the formulations was assayed by the CCK-8 test in the A549, CuFi-1, and NuLi-1 cell lines as models of pathological and healthy pulmonary epithelia (Figure 4).

Different concentrations of OLE ranging from 7.2 to 462.5 µM and its equivalent amount in NLCs were tested after 24 h of incubation. Equivalent concentrations of empty NLCs were also assayed as the control. Cell viability values >70% are considered as “no toxicity” [67] and thus, we assumed that NLCs as well as OLE were biocompatible with the tested lung epithelial cell lines. It is worth noting that NLC-empty did not shown any toxic effect, confirming the biocompatibility of the excipients of the NLCs (Supplementary Materials, Section S2). This finding is in accordance with other reports that demonstrated the good tolerability of lipid nanoparticles in lung epithelial cell lines [30,68,69]. Considering these results, OLE concentrations from 115.6 to 462 μM and their equivalent amount in NLC-OLE50 were chosen for the following antioxidant activity studies.

#### 3.3.2. Antioxidant Activity of Nanostructured Lipid Carriers (NLCs) in Lung Epithelial Cells: CAA Assay

The oxidative stress pathway has gained special attention as a novel target for the treatment of a number of diseases. Particularly within the lungs, oxidative stress has been correlated with lung cancerogenesis mechanisms as well as cystic fibrosis progression among others, in which natural antioxidants have been proposed as promising candidates [1,3,4,5,6]. Our optimized NLC-OLE50 had been demonstrated, by chemical analysis, to improve the radical scavenging power of OLE. Moreover, the size of our NLCs fell within the favorable range (100–200 nm) for an efficient cellular uptake, most likely to be taken up through the cell membranes by endocytosis mechanisms [70,71]. Beyond this, we have demonstrated a sustained release of the olive polyphenol from the lipid matrix within 24 h and thus, we assumed that OLE, which is protected inside the lipid core, might be progressively released to the cell, avoiding its rapid degradation and metabolism to other polyphenols (i.e., hydroxytyrosol, tyrosol, oleuropein aglycone), thus preserving or even improving its efficacy against oxidative stress. Altogether, this led us to hypothesize that NLC-OLE50 could be a promising carrier for OLE toward oxidative stress related injuries in lung epithelia. However, the lack of in vivo data about OLE efficacy in the lungs encouraged us to conduct a preliminary cell-based study to further confirm its effectiveness in lung cells.

**Figure 5:**
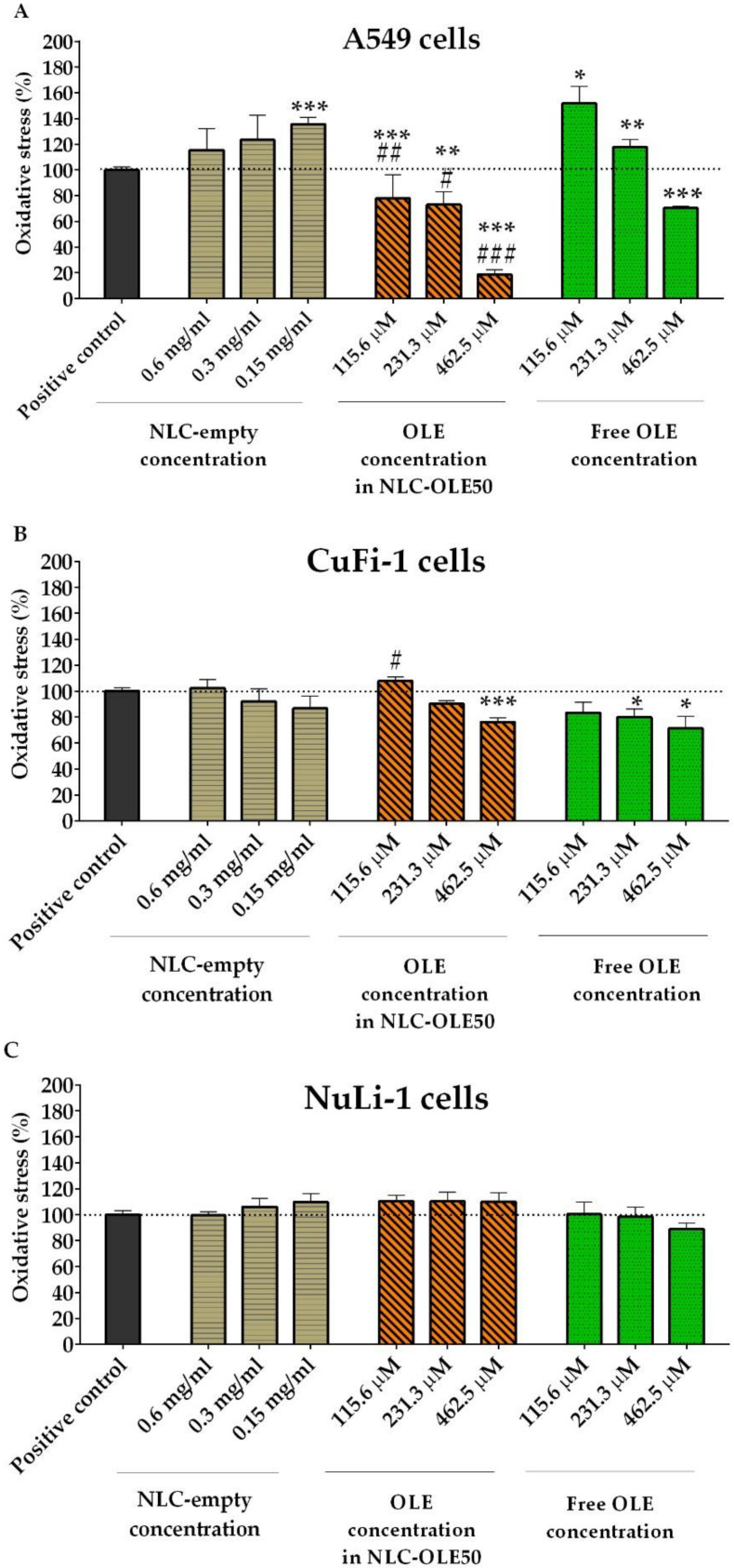
Antioxidant activity of free oleuropein (OLE), encapsulated OLE (NLC-OLE50), and empty nanoparticles (NLC-empty) in ABAP-stressed A549 (**A**), CuFi-1 (**B**), and NuLi-1 (**C**) cells. The mean percentages of DCF fluorescence, as a measure of oxidative stress, are shown in relation to the positive control (set to 100%). Results shown are means ± SEM of at least three independent experiments. * *p* < 0.05, ** *p* < 0.01, *** *p* < 0.001 vs. positive control; # *p* < 0.05, ## *p* < 0.01, ### *p* < 0.001 vs. free OLE.

Given this scenario, the radical scavenging activity of NLC-OLE50 and OLE was measured in ABAP-stressed A549, CuFi-1, and NuLi-1 as human-lung epithelial cell models, after 24 h of exposure to the mentioned treatments. The morphology of the cells was always checked to ensure that the induced oxidative damage did not cause cell death. Obtained results are displayed in Figure 5 as the percentage of intracellular ROS levels compared to the positive control (~100% of ROS levels).

Following our hypothesis, NLC-OLE50 clearly alleviated the oxidative stress status of A549 cells, which was found to be dose-dependent (Figure 5A). It should be noted that non-encapsulated OLE was only able to reduce ROS at the highest concentration tested (462.5 µM, *p* < 0.001) and, most importantly, it exerted prooxidant effects at 115 µM (*p* < 0.05) and 231 µM (*p* < 0.01). This result is in line with other authors who have demonstrated the prooxidant activity of OLE in some specific cancer cell lines of human breast cancer [72,73] and hepatocarcinoma [74]. However, there is still a lack of studies to further justify these findings. Therefore, we assumed that NLC-OLE50 significantly enhanced the antioxidant power of OLE in the A549 cell line, and this finding might be ascribed to a better permeability across cell membranes of the NLCs as well as their well-known higher uptake by the cellular model [65,75,76]. For the CuFi-1 human cells, NLC-OLE50 was demonstrated to preserve the moderate antioxidant power of OLE. Similarly, Hatahet et al. showed that the encapsulation of quercetin into NLCs did not improve its antioxidant activity, but preserved it in acute monocytic leukemia cells [77]. Evidence suggests that the misfolded CFTR protein can be modulated through antioxidant and prooxidant effects in cell environment and thus, we assumed that NLC-OLE50 and OLE might have interacted with the misfolded CFTR protein in CuFi-1 cells [1,78,79]. Unexpectedly, with regard to healthy epithelial cells (NuLi-1), neither free nor encapsulated OLE exerted any effect against ABAP produced peroxyl radical reaction, which could be ascribed to the inherent capacity of normal airways to cope with oxidative stress by themselves [79].

Given the aforementioned, the cellular antioxidant effects of NLC-OLE50 clearly depend on the studied cell line, which could be correlated with their different cellular uptake mechanisms. Nanoparticles are generally thought to be internalized through endocytosis mechanisms (i.e., phagocytosis, pinocytosis, clathrin-mediated, and caveolae/raft-mediated transports). Previous studies from our group demonstrated that the composition of the nanoparticles together with the type of cell can determine the predominant endocytosis mechanisms for their uptake and intracellular distribution, which ultimately affect the delivery of the active compound and the efficacy of the formulation [80,81,82]. These findings are in line with other authors who have demonstrated that the uptake of nanospheres by A549 cells could occur by clathrin-mediated endocytosis (PLGA and chitosan-PLGA nanospheres, 100 nm) as well as by the caveolae/raft-dependent transport (wheat germ agglutinin-conjugated PLGA nanospheres, 200 nm) [83]. On the other hand, evidence also suggests that depending on the size, shape, and surface charge of the nanoparticles, a particular cellular internalization route may be preferred over others. From the scarce data available about nanoparticle efficacy in cystic fibrosis cells, we found that authors have given special attention to the surface chemistry of the NLCs as an important influence factor for their cellular uptake and thus, one possibility of enhancing our NLC activity in CuFi-1 cells could be the modification of their superficial charge. However, whilst some authors have suggested that decreasing the surface charge of nanoparticles up to −50 mV could enhance their cellular uptake (CuFi-1 cells) [84], others have postulated that positive superficial charge is required to promote their internalization (CFBE41o-cells) [85] and thus it is highlighted again that special attention should be given not only to particle superficial charge, but also to the cell line and its predominant endocytosis mechanisms of transport. Similarly, the A549 cells were also shown to be more sensitive to OLE activity compared to the CuFi-1 and NuLi-1 cells. Since passive diffusion through cellular membranes is almost similar for all cells, scientific evidence suggests that differences in the active membrane transports are likely to be responsible for the differences observed in the CAA results between cell lines. Accordingly, the glucose moiety of OLE is known to interact with the membrane glucose transporter proteins (GLUT), allowing its diffusion into the cells. It is known that GUT is overexpressed in A549 cell membranes and thus we assumed that a higher uptake of OLE could have occurred in these cells, which ultimately might have led to a more pronounced intracellular antioxidant effect compared to the other cell lines [86]. A similar phenomenon has been found for the natural flavonoids quercetin, catechin, and epicatechin, which had superior antioxidant activities in Caco-2 compared to the HepG2 cell lines, probably due to a higher accumulation in Caco-2 cells [87,88]. However, little data are available about the role of transporters in cellular accumulation and evidence for individual phenolic compounds remains at a basic level. Finally, it should be noted that NLC-empty did not show any effect in the tested cell lines with the exception of A549 cells, which at the highest tested concentration seemed to increase ROS generation but without toxic effects.

All in all, the preserved antioxidant activity of OLE in NLCs holds great promise for transporting this natural polyphenol to the lung epithelia but further studies should be conducted to elucidate their internalization mechanism and intracellular trafficking processes in the proposed cells, which can be modulated by optimizing the formulations toward more efficient nanocarriers.

## 4. Conclusions

Whilst a number of preclinical data have revealed the therapeutic properties of olive polyphenols as plant extracts or pure synthetic molecules, scarce studies are available concerning isolated OLE and more specifically, naturally obtained OLE. In this work, OLE, a natural antioxidant with poor stability and compromised bioavailability, was successfully formulated in highly biocompatible NLCs. Optimized nanoparticles exhibited a mean size of 150 nm, demonstrated to be effective in loading up to 50% (*w/w*) of OLE with an encapsulation efficiency of 99.12%, a sustained release kinetic from the lipid core and enhanced antioxidant power was proven in the DPPH assay. Moreover, the rapid and simple proposed formulation process avoided the use of organic solvents and OLE loading was significantly higher compared to the other reported methods [25,27,28,89]. NLCs were found to be biocompatible in three lung epithelial cell lines, indicating their safety for lung administration. Interestingly, NLCs were shown to enhance and maintain the OLE protection effect against oxidative stress in lung cancer and cystic fibrosis cells, respectively, and thus, they could hold great promise for transporting this natural polyphenol to the lung epithelial cells. Nonetheless, further formulation studies should be conducted to obtain an adequate final formulation for pulmonary administration of NLC-OLE50 (i.e., dry powder for inhalation) [90,91,92].

## Figures and Tables

**Table 1:** Physico-chemical characterization of the developed nanoformulations with different solid lipid (Precirol ATO 5):liquid lipid (olive oil) ratio in the lipid core.

Formulation Code	Lipid Core (Precirol:Olive Oil, % *w/w*)	Size (nm)	Z Potential (mV)
1	10:90	134.30 ± 5.23	−17.08 ± 1.20
2	20:80	129.62 ± 3.65	−17.03 ± 5.89
3	30:70	123.88 ± 2.43	−18.03 ± 4.01
4	40:60	121.73 ± 1.86	−17.66 ± 4.59
5	50:50	130.27 ± 4.55	−19.82 ± 2.84
6	60:40	141.69 ± 11.43	−21.64 ± 3.72
7	70:30	152.76 ± 27.17	−25.34 ± 4.52
8	80:20	148.50 ± 0.03	−26.16 ± 4.93
9	90:10	158.44 ± 7.57	−19.16 ± 2.52

**Table 2:** Oleuropein (OLE) release parameters of different kinetic models.

Sample	Kinetic Models														
	Zero Order		First Order		Higuchi		Baker–Londslade		Hixson–Crowel		Hopfenger		Korsmeyer–Peppas		
	**r^2^**	**k**	**r^2^**	**k**	**r^2^**	**k**	**r^2^**	**k**	**r^2^**	**k**	**r^2^**	**k**	**r^2^**	**kp**	**n**
NLC-OLE30	−0.100	3.035	0.551	0.072	0.776	14.87	0.844	0.005	0.871	0.02	0.776	0	**0.977**	21.29	0.46
NLC-OLE40	−0.157	0	0.741	0.113	0.723	0.019	0.847	0.009	0.585	0.029	0.596	0.020	**0.998**	26.59	0.52
NLC-OLE50	0.150	3.680	0.836	0.108	0.840	17.89	0.947	0.009	0.708	0.028	0.733	0.022	**0.999**	20.61	0.59

r^2^, determination coefficient; k, release kinetic constant; kp, Korsmeyer–Peppas constant; n, diffusion release exponent.

**Table 3:** Thermal properties of Precirol and nanoparticles (NLC-empty, NLC-OLE30, NLC-OLE40, and NLC-OLE50).

Sample	Melting Point (°C)	Onset (°C)	Endset (°C)	Enthalpy (J/g)	CI (%)
Precirol^®^ ATO5	56.00	50.12	62.97	−150.56	100.00
NLC-empty	51.05	40.18	61.28	−90.21	59.92
NLC-OLE30	50.47	41.71	61.34	−56.22	37.34
NLC-OLE40	50.16	43.27	61.38	−57.15	37.96
NLC-OLE50	50.88	40.86	62.67	−63.09	41.90

CI (%): Crystallinity index.

## Supplementary Materials

The following are available online at https://www.mdpi.com/1999-4923/12/5/429/s1, Section S1: Nanoparticle morphology and success in freeze-drying process of blank nanoparticles. Section S2: Cell viability assay of NLC-empty.
