# Supplementary material for: Encapsulation of Oleuropein in Nanostructured Lipid Carriers: Biocompatibility and Antioxidant Efficacy in Lung Epithelial Cells
Source: Pharmaceutics. 2020 May 6;12(5):429. doi: 10.3390/pharmaceutics12050429 (PMC7285197; doi:10.3390/pharmaceutics12050429)
Supplement: Supplementary file 1 [file pharmaceutics-12-00429-s001.pdf]

pharmaceutics
MDPI

Supplementary Materials: Encapsulation of Oleuropein in Nanostructured Lipid Carriers: Biocompatibility and Antioxidant Efficacy in Lung Epithelial Cells

Amaia Huguet-Casquero, Maria Moreno-Sastre, Tania Belén Lopez-Méndez, Eusebio Gainza and Jose Luis Pedraz

S1. Nanoparticle Morphology and Success in Freeze-Drying Process of Blank Nanoparticles

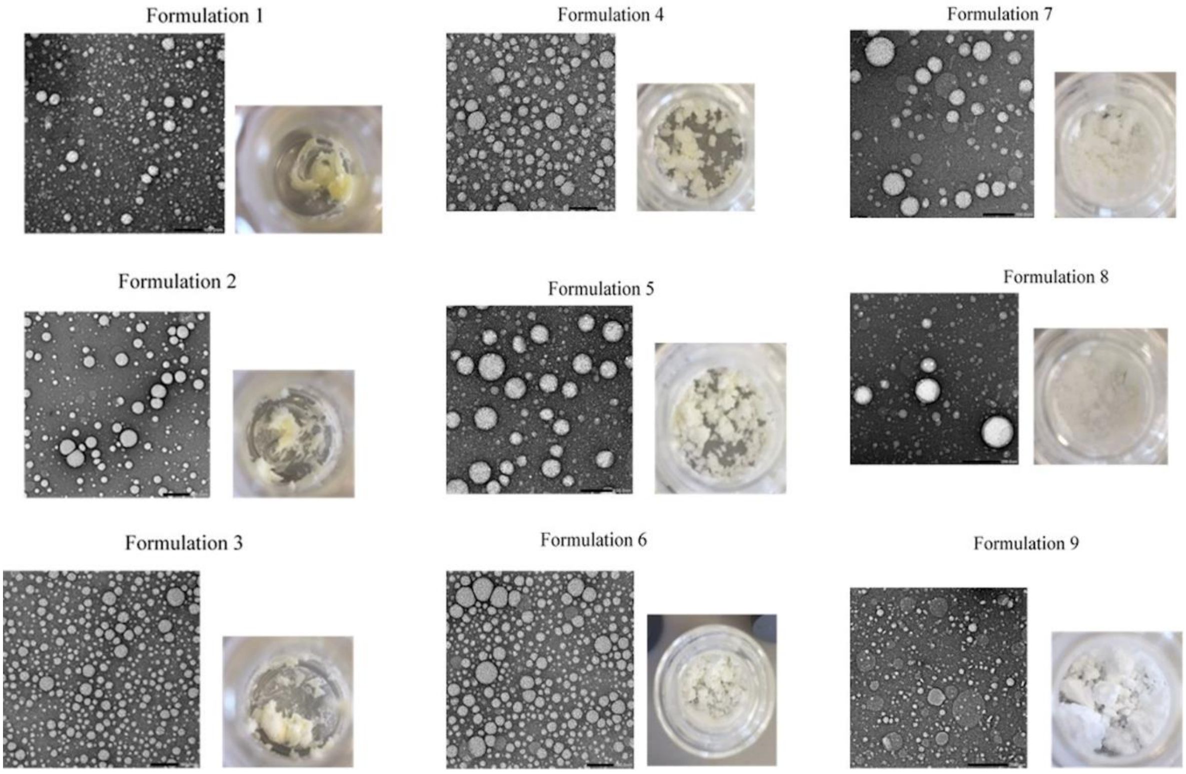

Figure S1. TEM micrographs and photographs of freeze-dried nanoformulations with different solid-liquid lipid ratios. The scale bar of TEM micrographs (bottom right) indicates 100 nm.

S2. Cell Viability Assay of NLC-Empty

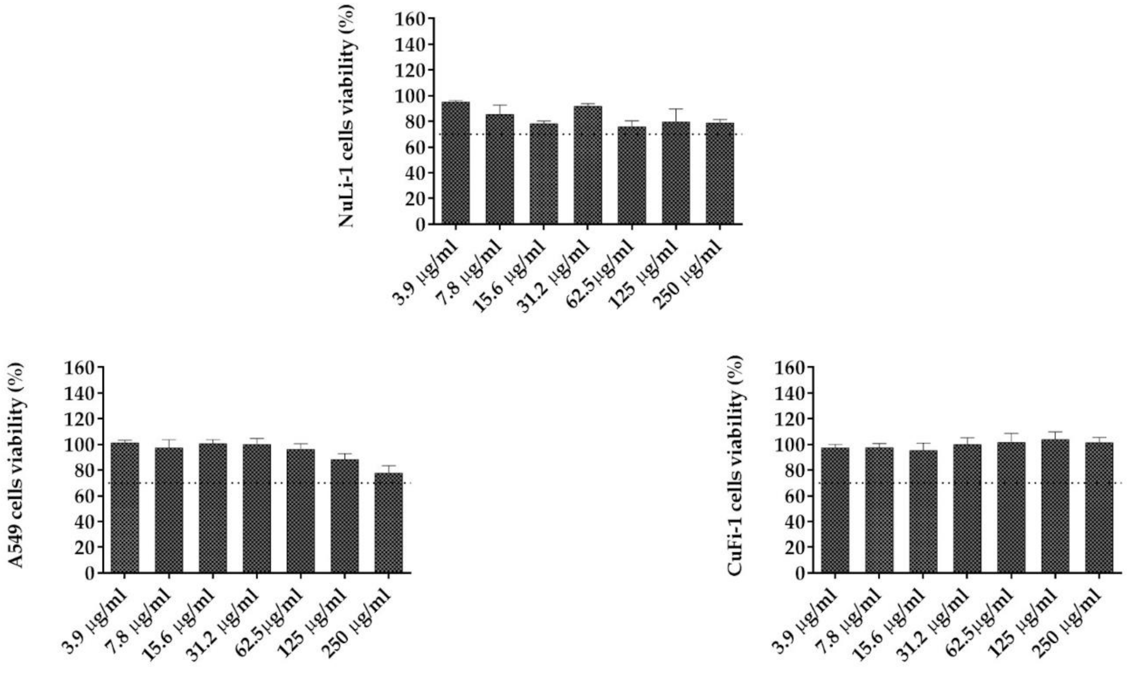

Figure S2. Effect of NLC-empty at different concentrations on the viability of NuLi (upper), A549 (bottom-left) and CuFi-1 (bottom-right) cells. Results are given as the mean % of living cells compared to the control ± SD, n = 3.
